# Exploring the molecular link between arecoline exposure and prostate cancer-related alterations: integrative evidence from network toxicology, single-cell transcriptomics, molecular simulation, and experimental validation

**DOI:** 10.3389/fphar.2026.1851696

**Published:** 2026-06-08

**Authors:** Xinyao Zhu, Haixia Liu, Zhiqiang Zeng, Lunhong Zou, Yubo Zhou, Tao Zhou

**Affiliations:** 1 Department of Urology, Santai Hospital Affiliated to North Sichuan Medical College, Mianyang, Sichuan, China; 2 Department of Urology, Affiliated Hospital of Southwest Medical University, Luzhou, Sichuan, China

**Keywords:** androgen receptor, arecoline, environmental toxicology, food-related exposure, network toxicology, prostate cancer

## Abstract

Arecoline, the major alkaloid of areca nut, is a common exposure in chewing products, but its relationship with prostate cancer (PCa) is unclear. We integrated network toxicology, bulk RNA machine learning, single-cell transcriptomics, molecular simulation, and *in vitro* validation to prioritize candidate molecular nodes potentially linking arecoline exposure to PCa-related alterations. Potential arecoline targets were intersected with PCa-related genes, followed by protein–protein interaction and enrichment analyses. Candidate genes were prioritized using TCGA-PRAD and external GEO cohorts, then refined in GWAS-relevant epithelial subpopulations from GSE141445. AR was further evaluated by the Human Protein Atlas, molecular docking, molecular dynamics, RT-qPCR, and Western blotting. We identified 97 overlapping targets enriched mainly in apoptosis-, p53-, and MAPK-related pathways. The optimal bulk RNA model showed good external performance (AUC = 0.956). Integrative analyses identified androgen receptor (AR) as the only consensus core gene. AR-high epithelial cells showed increased androgen response, mTORC1 signaling, and MYC targets. Molecular simulation provided computational support for structural compatibility between arecoline and AR, while *in vitro* experiments showed increased AR mRNA and protein expression after arecoline treatment in LNCaP cells. These findings suggest AR as a plausible candidate molecular node linking arecoline exposure to PCa-related molecular alterations and provide a hypothesis-generating framework for future mechanistic and exposure-focused studies.

## 1 Introduction

Prostate cancer (PCa) is one of the most frequently diagnosed malignancies in men worldwide and remains a leading cause of cancer-related mortality (Bray et al., 2024). Although prostate-specific antigen screening, radical surgery, radiotherapy, and androgen deprivation therapy have improved clinical outcomes in selected patients, advanced disease—particularly castration-resistant prostate cancer (CRPC)—continues to pose major therapeutic challenges (Bray et al., 2024; Raychaudhuri et al., 2025). Among the molecular determinants that drive PCa development and progression, the androgen receptor (AR) occupies a central position (Li et al., 2025). AR not only regulates normal prostate development and homeostasis, but also plays critical roles in tumor initiation, sustained proliferation, metastatic progression, and therapeutic resistance (Li et al., 2025; Watson et al., 2015; Balk, 2014; Bernard-Tessier et al., 2025). Accordingly, clarifying the upstream factors that contribute to aberrant AR activation is of considerable importance for understanding PCa pathogenesis. In particular, increasing attention has been directed toward exogenous toxicant-related modulators arising from environmental and food-related exposure scenarios, as these factors may influence hormone-responsive oncogenic programs in ways that remain insufficiently understood (Modica et al., 2023; Lacouture et al., 2022; Feijo et al., 2025).

Arecoline is one of the major alkaloids in areca nut and represents a principal bioactive component responsible for many of its biological effects (Huang et al., 2024). Because areca-derived products are consumed or chewed by large populations in certain regions, arecoline has relevance not only as an environmentally encountered exogenous compound, but also as a toxicologically meaningful agent in oral and food-related exposure contexts (Mehrtash et al., 2017; Gupta and Warnakulasuriya, 2002; Arora and Squier, 2019). Previous studies have more extensively documented the carcinogenic or tumor-promoting effects of arecoline in oral cancer and certain gastrointestinal malignancies (Wang Z. et al., 2024; Ko et al., 2023; Guha et al., 2014; Akhtar, 2013; Senevirathna et al., 2023; Warnakulasuriya and Chen, 2022). By comparison, its potential involvement in prostate cancer has not been systematically investigated. Existing evidence suggests that arecoline may affect both normal prostate cells and prostate cancer cells through mechanisms involving reactive oxygen species (ROS) and cell-cycle regulation, implying that the prostate may represent a biologically relevant target organ of arecoline exposure (Shih et al., 2020; Wang T. H. et al., 2024). However, whether arecoline participates in the dysregulation of PCa-associated molecular programs, particularly those related to AR signaling, remains largely unclear. This gap provides the main rationale for focusing on PCa in the present study. Although the best-established carcinogenic relevance of arecoline remains in oral and upper aerodigestive tract malignancies, several considerations support the biological plausibility of investigating its potential relationship with PCa-related molecular alterations. First, the prostate is a hormone-responsive organ that is sensitive to endocrine-disrupting and toxicant-related molecular perturbations. Second, AR is a central regulator of PCa biology and may serve as a molecular interface through which exogenous compounds influence hormone-responsive transcriptional programs. Third, previous experimental observations in prostate-related models suggest that arecoline can affect ROS production, cell-cycle regulation, and prostate tissue or cell phenotypes. Therefore, the possible association between arecoline exposure and AR-related PCa molecular programs represents a biologically plausible but insufficiently explored question.

With the rapid development of systems biology and transcriptomics, integrative analytical strategies have provided new opportunities to investigate the complex relationships between exogenous exposures and disease. Network toxicology can identify candidate targets at the intersection of compound-associated gene networks and disease-related molecular programs, thereby offering a useful framework for studying toxicant–disease associations (Valls-Margar et al., 2023). Bulk RNA-based machine learning is valuable for prioritizing robust candidate genes at the tissue level, whereas single-cell RNA sequencing enables these candidates to be localized to specific cellular subpopulations within the tumor microenvironment (Stuart et al., 2019). Furthermore, when single-cell transcriptomic analysis is integrated with genome-wide association study (GWAS)-guided prioritization of disease-relevant cell populations, the reliability of candidate target identification can be improved at the tissue, cellular, and genetic levels (Lai et al., 2024). Such a multi-layered framework is particularly suitable for toxicological studies in which the biological effects of environmental or food-related exposures may be heterogeneous, context-dependent, and distributed across multiple molecular levels.

Accordingly, the present study was designed as an exploratory and hypothesis-generating investigation integrating network toxicology, bulk RNA machine learning, single-cell transcriptomic analysis, SeismicGWAS, molecular docking, molecular dynamics simulation, and *in vitro* experimental validation. Rather than establishing a causal relationship between arecoline exposure and PCa, our aim was to prioritize candidate molecular nodes that may connect arecoline-related target networks with PCa-associated transcriptional and cellular states. We further sought to characterize the expression patterns, cell-subpopulation distribution, and preliminary biological relevance of these candidate nodes. By doing so, this study provides a multi-layered toxicological framework for future mechanistic and exposure-focused investigations into how orally encountered exogenous compounds may influence AR-related molecular programs in hormone-responsive malignancies.

## 2 Materials and methods

### 2.1 Acquisition of the chemical structure and basic information of arecoline

The chemical information of arecoline was retrieved from the PubChem database, including its two-dimensional and three-dimensional structures, canonical SMILES string, molecular formula, molecular weight, and other basic physicochemical parameters. These structural data were used for subsequent target prediction, molecular docking, and molecular dynamics simulation analyses.

### 2.2 Prediction of potential targets of arecoline

To comprehensively identify potential human targets of arecoline, three online databases—CTD, SwissTargetPrediction, and SEA—were jointly used for target prediction. Specifically, the keyword “arecoline” was used in the CTD database to retrieve related genes/proteins, while the SMILES string of arecoline was submitted to SwissTargetPrediction and SEA with the species restricted to *Homo sapiens*. The inclusion criteria for arecoline targets were as follows: targets had to be associated with arecoline in at least one of the above databases, mapped to *H. sapiens*, and successfully converted to an official gene symbol. Targets were excluded if they were non-human entries, lacked valid gene-symbol annotation, or represented duplicated records after symbol standardization. The results from the three databases were then merged, standardized to official gene symbols using the UniProt database, and deduplicated, yielding the final set of potential arecoline targets. Because these resources include predicted, curated, and literature-derived associations, the database-derived targets were regarded as a hypothesis-generating candidate target space rather than experimentally confirmed direct targets of arecoline. Therefore, subsequent analyses were designed to prioritize candidate genes through multi-step filtering and cross-level evaluation.

### 2.3 Collection of prostate cancer-related genes

PCa-related genes were retrieved from the GeneCards and OMIM databases using the keyword “Prostate cancer.” In GeneCards, genes with a relevance score >10 were retained to improve the confidence of disease-gene screening and reduce the inclusion of weakly associated entries. PCa-related genes reported in OMIM were extracted as curated disease-associated genes. Genes were included if they were associated with prostate cancer in either GeneCards after relevance-score filtering or OMIM, and excluded if they lacked valid official gene-symbol annotation or represented duplicated entries after merging. The two gene sets were standardized to official gene symbols, combined, and deduplicated to construct the final prostate cancer-associated gene set.

### 2.4 Screening of overlapping targets, PPI network construction, and functional enrichment analysis

#### 2.4.1 Screening of overlapping targets and construction of the PPI network

R software was used to identify the overlap between potential arecoline targets and PCa-associated genes. The resulting common candidate genes were then uploaded to the STRING database to construct a protein–protein interaction (PPI) network, with the species set to *H. sapiens* and the minimum interaction confidence score set to 0.400. After removal of disconnected nodes, the network was exported and imported into Cytoscape for visualization and topological analysis. The DMNC algorithm in the cytoHubba plugin was used to rank node importance and identify hub genes in the network.

#### 2.4.2 GO and KEGG enrichment analyses

GO and KEGG enrichment analyses were performed using the clusterProfiler and org. Hs.e.g.,.db packages in R. GO analysis included biological process (BP), cellular component (CC), and molecular function (MF) categories. All enrichment results were adjusted for multiple testing using the Benjamini–Hochberg method, and an adjusted P value <0.05 was considered statistically significant. Significant terms were visualized using ggplot2.

### 2.5 Construction and validation of machine learning-based diagnostic models at the bulk RNA level

#### 2.5.1 Data sources and preprocessing

Transcriptomic data from the TCGA-PRAD cohort were downloaded from the TCGA database and used as the training set. GSE46602 and GSE70768 were obtained from the GEO database and used as external validation cohorts. The TCGA-PRAD cohort was used exclusively for feature selection and model fitting, whereas GSE46602 and GSE70768 were not used during feature selection, model training, or threshold determination. To improve comparability across validation cohorts, common genes shared by all datasets were first identified. The two GEO validation datasets were then merged, and batch effects between GSE46602 and GSE70768 were corrected using the ComBat algorithm implemented in the sva package before model validation. The TCGA-PRAD training cohort and GEO validation cohorts were processed separately to minimize information leakage. PCA and hierarchical clustering were used to evaluate batch-correction performance.

#### 2.5.2 Feature input and data standardization

The overlapping genes identified by network toxicology were used as candidate features. Zero-variance and missing-value features were removed during preprocessing. The training and validation sets were then independently standardized using Z-score normalization.

#### 2.5.3 Construction of 130 machine learning algorithm combinations

A two-stage modeling strategy was used. In the first stage, feature selection was performed using LASSO regression, Ridge regression, elastic net, stepwise logistic regression, random forest (RF), and gradient boosting machine (GBM). In the second stage, the selected feature subsets were used as input for classifiers including support vector machine (SVM), linear discriminant analysis (LDA), GBM, partial least squares logistic regression (plsRglm), random survival forest (RSF), naïve Bayes, XGBoost, and multivariable logistic regression. Different feature selection methods were combined with different classifiers to generate a total of 130 machine learning models. RF parameters were set as ntree = 1000 and nodesize = 5; GBM parameters were set as n. trees = 10,000, shrinkage = 0.001, and interaction. depth = 3, with the optimal number of iterations determined by cross-validation; for XGBoost, the optimal nrounds was determined by cross-validation.

#### 2.5.4 Model performance evaluation and selection of the optimal model

The area under the receiver operating characteristic curve (AUC) was used as the primary performance metric. AUC values were calculated for each model in the training and external validation sets, and models were compared based on their overall performance across datasets. The algorithm combination with the best integrated performance was selected as the optimal model. ROC curves were then generated for the optimal model, and the corresponding AUC and 95% confidence interval were calculated. In addition to AUC, standard classification metrics, including accuracy, precision, recall, and F1-score, were calculated for the top-performing model in both the training and external validation datasets. The classification threshold was determined using the TCGA-PRAD training cohort and then applied unchanged to the external GEO validation cohorts to avoid threshold optimization in the validation datasets. The machine learning framework was used for candidate gene prioritization rather than for developing a clinically deployable diagnostic model or an exposure-related risk prediction model. Although external GEO cohorts were used for performance evaluation, a formal nested cross-validation framework was not applied; therefore, model performance was interpreted cautiously.

### 2.6 Single-cell transcriptomic analysis and identification of key cell subpopulations

#### 2.6.1 Single-cell data source

The prostate cancer single-cell RNA sequencing dataset GSE141445 was downloaded from the GEO database. Summary statistics of the prostate cancer GWAS dataset (ieu-b-85) were obtained from the IEU GWAS database for subsequent identification of disease-related cell subpopulations.

#### 2.6.2 Quality control

Seurat was used for quality control of the raw single-cell expression matrix. Seurat objects were initially generated with min. cells = 5 and min. features = 300. The proportions of mitochondrial and ribosomal genes were calculated for each cell using PercentageFeatureSet. Quality-control visualization was performed using violin plots and feature scatter plots of nFeature_RNA, nCount_RNA, mitochondrial gene proportion, and ribosomal gene proportion. Cells were retained if they met the following criteria: UMI count ≥1000, number of detected genes between 200 and 10,000, mitochondrial gene proportion ≤20%, and ribosomal gene proportion ≤20%. These filtering steps were used to remove low-quality cells, empty droplets, and potential doublet-containing cells.

#### 2.6.3 Data normalization, batch correction, dimensionality reduction, and clustering

The filtered single-cell expression matrix was normalized using the LogNormalize method implemented in Seurat, with a scale factor of 10,000. Highly variable genes were identified using the FindVariableFeatures function with the vst method, and the top 2,000 variable genes were retained. The data were then scaled using ScaleData, and principal component analysis (PCA) was performed based on the variable genes. Harmony was subsequently used for batch correction with sample type as the grouping variable to reduce the effects of sample origin on clustering results. A nearest-neighbor graph was constructed using the first 10 principal components, and graph-based clustering was performed using FindClusters. Different resolutions from 0.2 to 1.2 were explored using clustree, and a final resolution of 0.7 was selected for downstream analysis. UMAP was used for visualization.

#### 2.6.4 Cell type annotation

Marker genes for each cluster were identified using FindAllMarkers, and cell types were manually annotated based on canonical markers and published references. Representative markers included epithelial cells (EPCAM, KRT18, KRT19), endothelial cells (PECAM1, VWF, ACKR1), fibroblasts (COL1A2, TAGLN, ACTA2), myeloid cells (CD14, FCGR3A, CD163), T cells (CD3D, CD3E, GZMA), B cells (CD79A, MS4A1, DERL3), and mast cells (TPSAB1, TPSB2, MS4A2). This procedure yielded the major cell types and their distribution within prostate cancer tissues.

#### 2.6.5 Identification of disease-associated cell subpopulations based on SeismicGWAS

The SeismicGWAS framework was applied to integrate single-cell transcriptomic data with GWAS data in order to identify cell subpopulations most strongly associated with prostate cancer genetic risk. First, MAGMA was used to aggregate variant-level GWAS signals to the gene level. Next, gene expression specificity scores across annotated cell subpopulations were calculated, and gene symbols were converted to Entrez IDs for matching with MAGMA results. Finally, SeismicGWAS was used to estimate the strength of association between each cell subpopulation and prostate cancer genetic susceptibility, thereby identifying key disease-associated cell subpopulations.

### 2.7 Machine learning analysis at the single-cell level

#### 2.7.1 Metacell construction

To reduce the influence of high sparsity and technical noise in single-cell data on machine learning analyses, the hdWGCNA package was used to construct metacells. Neighboring cells were aggregated into pseudo-bulk units, and the resulting expression matrix was normalized for downstream analysis.

#### 2.7.2 Feature gene matching and filtering

The overlapping genes identified by network toxicology were used as input features and matched to the metacell expression matrix. Genes with excessively low mean expression or insufficient variance were removed, retaining only features with stable expression and adequate discriminative potential.

#### 2.7.3 Evaluation of 45 machine learning algorithm combinations

A total of 45 machine learning algorithm combinations were constructed within the key epithelial cell subpopulations identified by SeismicGWAS. Specifically, random forest, XGBoost, LASSO regression, Ridge regression, elastic net, radial and linear kernel SVM, GBM, and naïve Bayes were used for feature selection, and the resulting feature sets were combined with RF, radial SVM, XGBoost, elastic net, and naïve Bayes classifiers. Model performance was evaluated using repeated 10-fold cross-validation with 10 repeats, and cross-validated AUC (CV-AUC) was used as the primary performance metric. Similar to the bulk RNA analysis, the single-cell machine learning procedure was used to prioritize candidate genes within disease-relevant epithelial subpopulations rather than to establish a clinically applicable prediction model.

#### 2.7.4 Consensus gene selection by integrated voting

An elite model threshold of CV-AUC ≥0.8 was defined, and only models meeting this threshold were included in subsequent voting. For each elite model, the selected or top-ranked genes were recorded, and the frequency of each gene across elite models was counted and weighted by the corresponding CV-AUC values. Consensus candidate genes at the single-cell level were then selected based on a predefined minimum voting ratio. This voting procedure was used to identify genes repeatedly prioritized across multiple algorithmic settings, rather than to provide independent validation of predictive performance.

#### 2.7.5 SHAP-based interpretability analysis

To improve model interpretability, a random forest model combined with SHAP (SHapley Additive exPlanations) analysis was used to evaluate the relative contribution of consensus candidate genes to model output. SHAP beeswarm and bar plots were generated to show the direction and magnitude of gene contributions. The SHAP analysis was interpreted as an explanatory tool for candidate prioritization rather than as independent validation of gene function.

### 2.8 GSVA of AR-high and AR-low cells within key epithelial subpopulations

Within the key epithelial cell subpopulations identified by SeismicGWAS, cells were divided into AR-high and AR-low groups according to AR expression levels. Gene set variation analysis (GSVA) was then performed using the Hallmark gene sets from the MSigDB database to compare pathway activity differences between the two groups. GSVA scores were standardized and visualized using a heatmap.

### 2.9 Validation of protein expression using the HPA database

To validate the protein-level expression pattern of the candidate core gene, AR immunohistochemistry data were retrieved from the Human Protein Atlas (HPA) database. Staining intensity and localization patterns in normal prostate tissue and prostate cancer tissue were compared to evaluate the differential expression of AR in clinical tissue samples.

### 2.10 Molecular docking, two-dimensional interaction analysis, and molecular dynamics simulation

#### 2.10.1 Molecular docking

Molecular docking was performed using AutoDock Vina 1.1.2. The crystal structure of human AR was obtained from the Protein Data Bank (PDB ID: 1XOW), and the three-dimensional structure of arecoline was obtained from PubChem. Arecoline was energy-minimized before docking. Receptor and ligand preparation was performed using AutoDockTools, including removal of water molecules and non-protein molecules where appropriate, addition of polar hydrogens, charge assignment, and conversion to PDBQT format.

A blind docking strategy was adopted to explore the potential binding mode of arecoline with AR without predefining a specific binding pocket. The docking grid was set to cover the AR ligand-binding region, with the grid center set at X = 21.994, Y = 5.377, and Z = 10.944. The grid dimensions were 114 × 90 × 110 points with a spacing of 0.4417 Å, corresponding to an approximate search box of 50.35 × 39.75 × 48.58 Å. The default AutoDock Vina empirical scoring function was used to estimate binding affinity, and docking poses were ranked according to predicted binding energy. A total of 20 docking poses were generated, and the conformation with the lowest binding energy and reasonable interaction geometry was selected for subsequent two-dimensional interaction analysis and molecular dynamics simulation.

Because no experimentally resolved arecoline–AR complex is available and redocking validation was not performed in this study, the docking results were interpreted as exploratory structural modeling evidence rather than definitive proof of direct arecoline–AR binding.

#### 2.10.2 Two-dimensional interaction visualization

To further characterize the binding pattern between arecoline and AR, the optimal docking conformation was imported into Discovery Studio for two-dimensional interaction analysis. Non-covalent interactions, including carbon hydrogen bonds, Pi-Sigma interactions, Alkyl/Pi-Alkyl interactions, and van der Waals contacts, were identified to determine key interacting residues and interaction types.

#### 2.10.3 Molecular dynamics simulation and binding free energy calculation

Molecular dynamics simulations were performed using GROMACS 2023.2. The AR protein was parameterized using the AMBER99SB force field, and the arecoline ligand topology was generated using ACPYPE based on the GAFF force field. The arecoline–AR complex obtained from molecular docking was placed in a cubic box with a minimum distance of 1.0 nm from the box edge and solvated using the TIP3P water model. Counterions were added to neutralize the system.

Energy minimization was performed in two sequential steps using the steepest descent and conjugate gradient algorithms, respectively, with a maximum of 50,000 steps and an energy convergence criterion of 1000 kJ/mol/nm. The system was then equilibrated under NVT and NPT ensembles. Both NVT and NPT equilibration were performed for 100,000 steps with a time step of 2 fs. During equilibration, position restraints were applied to the protein–ligand complex. Temperature coupling was performed using the V-rescale thermostat, and pressure coupling during NPT equilibration was performed using the Parrinello–Rahman barostat.

A 100-ns production MD simulation was subsequently performed with a time step of 2 fs at 300 K and 1 bar. Long-range electrostatic interactions were treated using the particle mesh Ewald method, and covalent bonds involving hydrogen atoms were constrained using the LINCS algorithm. A Verlet cutoff scheme was used, with short-range electrostatic and van der Waals cutoffs set to 1.2 nm. RMSD, RMSF, radius of gyration, solvent-accessible surface area, hydrogen bond number, and free energy landscape were calculated to assess conformational stability. Binding free energy was estimated using the MM/PBSA method. Replicate MD simulations were not performed; therefore, the MD results were interpreted as supportive computational evidence rather than definitive proof of dynamic stability under all simulation conditions.

### 2.11 *In vitro* experimental validation

#### 2.11.1 Cell culture and treatment

The human prostate cancer cell line LNCaP was cultured in RPMI-1640 medium supplemented with 10% fetal bovine serum (FBS) and 1% penicillin–streptomycin under standard conditions (37 °C, 5% CO_2_). When cells reached appropriate density, arecoline treatment was initiated.

Arecoline working solutions were prepared in PBS. According to the experimental design, cells were treated with 0.2 mM and 0.4 mM arecoline for 24 h. The experimental groups were set as follows: Con, Con + PBS, Con +0.2 mM arecoline, and Con +0.4 mM arecoline. The PBS group served as the vehicle control. The concentrations of 0.2 and 0.4 mM arecoline correspond to approximately 31.0 and 62.1 μg/mL, respectively, based on the molecular weight of arecoline. These concentrations were selected with reference to previous *in vitro* studies in prostate cells and reported salivary arecoline levels during areca nut chewing (Shih et al., 2020; Cox et al., 2010). Nevertheless, because salivary exposure cannot be directly equated with systemic or prostate tissue exposure, these concentrations were used as exploratory *in vitro* exposure conditions rather than as direct estimates of physiological prostate exposure in humans.

#### 2.11.2 RNA extraction and RT-qPCR

After treatment, total RNA was extracted from each group of cells using TRIzol reagent. RNA concentration and purity were measured using a spectrophotometer, and equal amounts of RNA were reverse-transcribed into cDNA. Real-time quantitative PCR was then performed to determine AR mRNA expression, with β-actin used as the internal control. Primer sequences (5′–3′) were as follows: AR, forward AAG​ACG​CTT​CTA​CCA​GCT​CAC​CAA, reverse TCC​CAG​AAA​GGA​TCT​TGG​GCA​CTT; β-actin, forward GAT​GAG​ATT​GGC​ATG​GCT​TT, reverse CAC​CTT​CAC​CGG​TCC​AGT​TT. Relative gene expression was calculated using the 2^-ΔΔCt method. All experiments were independently repeated at least three times.

#### 2.11.3 Protein extraction and Western blotting

After treatment, total protein was extracted from each group using RIPA lysis buffer, and protein concentration was measured using the BCA assay. Equal amounts of protein were separated by SDS-PAGE and transferred onto PVDF membranes. After blocking, membranes were incubated with primary antibodies against AR (Proteintech, Cat No. 22089-1-AP, 1:10,000) and β-actin (Proteintech, Cat No. 66009-1-Ig, 1:20,000) as the loading control. After incubation with the corresponding HRP-conjugated secondary antibodies, signals were visualized using enhanced chemiluminescence and captured. Band intensities were quantified using ImageJ, and AR protein expression was normalized to β-actin.

### 2.12 Statistical analysis

Bioinformatic analyses were primarily conducted in R. For enrichment analyses, an adjusted P value <0.05 was considered statistically significant. Statistical analyses of RT-qPCR and Western blot data were performed using GraphPad Prism. All *in vitro* experiments were independently repeated at least three times. Data are presented as mean ± standard deviation (SD). Comparisons among multiple groups were performed using one-way ANOVA followed by Tukey’s multiple comparison test. A P value <0.05 was considered statistically significant.

## 3 Results

### 3.1 Screening of overlapping targets between arecoline and prostate cancer and functional enrichment analysis

**FIGURE 1:**
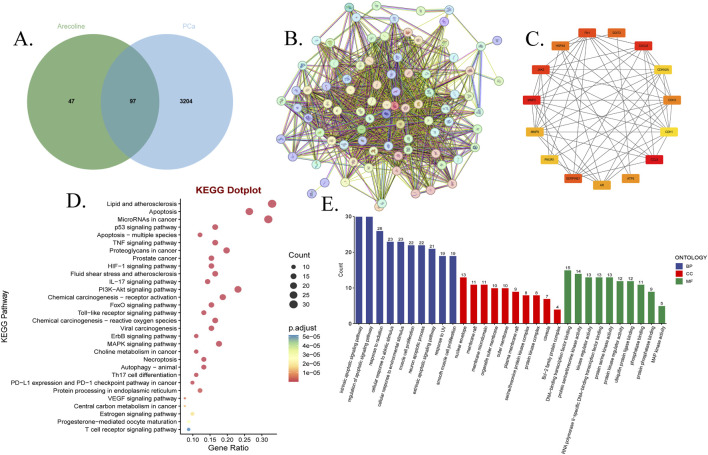
Screening of overlapping targets between arecoline and prostate cancer and functional enrichment analysis **(A)** Venn diagram showing the overlap between predicted arecoline targets and prostate cancer-related genes, yielding 97 common candidate targets **(B)** Protein–protein interaction (PPI) network of the 97 overlapping targets constructed using the STRING database **(C)** Hub gene network identified by the DMNC algorithm **(D)** KEGG enrichment analysis of the overlapping targets, showing significant enrichment in pathways including lipid and atherosclerosis, apoptosis, p53, TNF, HIF-1, IL-17, and MAPK signaling **(E)** GO enrichment analysis of the overlapping targets, including biological process (BP), cellular component (CC), and molecular function (MF).

The basic chemical structure of arecoline was first obtained from PubChem (Table 1). Potential arecoline-related targets were then predicted using the SEA, CTD, and SwissTargetPrediction databases, yielding 144 candidate human targets. Meanwhile, 3301 PCa-related genes were collected from the GeneCards and OMIM databases. Intersection analysis identified 97 common candidate targets, suggesting that these genes may constitute the molecular basis linking arecoline exposure to PCa-related biological processes (Figure 1A).

**TABLE 1:** Basic chemical and structural information of arecoline.

Name	SMILES structures	CAS	Molecular formula	Molecular weight
Arecoline	CN1CCC = C(C1)C(=O)OC	63-75-2	C8H13NO2	155.19 g/mol

The 97 overlapping targets were subsequently imported into STRING to construct a PPI network and visualized in Cytoscape. Based on the DMNC algorithm, AR, FN1, and MMP2 ranked among the top hub genes, indicating that they may occupy relatively important positions in the arecoline–PCa-associated molecular network (Figures 1B,C).

Functional enrichment analysis showed that these overlapping genes were mainly involved in classical signaling pathways, including apoptosis, p53, TNF, HIF-1, IL-17, and MAPK. GO analysis further suggested that these genes were associated with apoptosis regulation, stress response, and cell proliferation-related processes. Collectively, these enrichment results suggest that the overlapping targets are mainly involved in broad cancer- and stress-related biological processes, including tumor cell survival, stress responses, and inflammatory signaling networks. These pathways were interpreted as functional context for subsequent candidate screening rather than as evidence of arecoline-specific or PCa-specific mechanisms (Figures 1D,E).

### 3.2 Construction of machine learning models and candidate gene screening at the bulk RNA level

**FIGURE 2:**
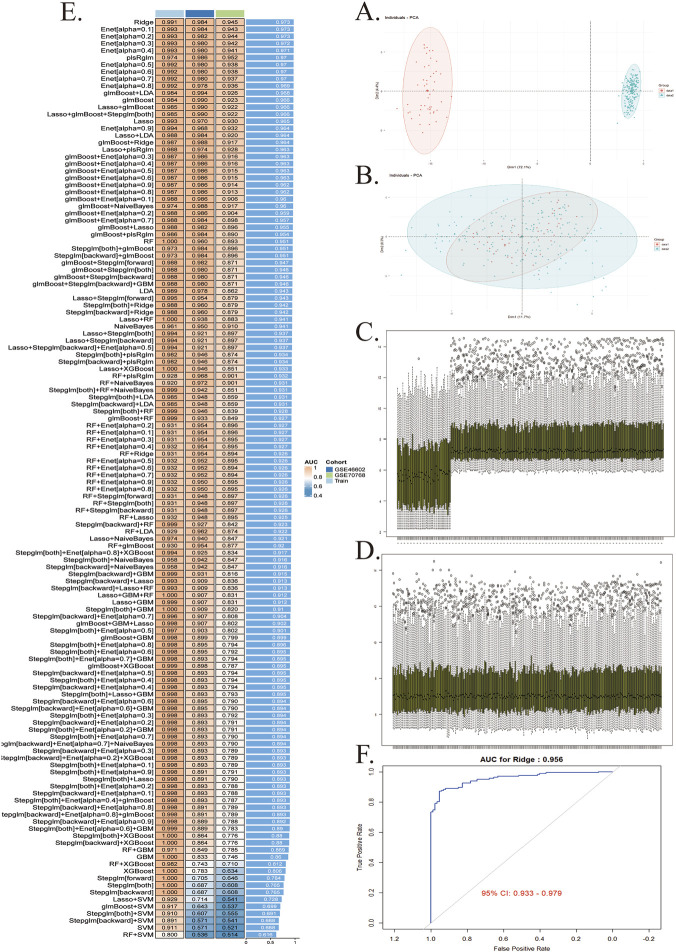
Construction of machine learning models and selection of the optimal model at the bulk RNA level **(A)** PCA plot before batch correction of GSE46602 and GSE70768 **(B)** PCA plot after batch correction of GSE46602 and GSE70768 **(C)** Boxplot of the expression matrix before batch correction **(D)** Boxplot of the expression matrix after batch correction **(E)** AUC heatmap of 130 machine learning algorithm combinations constructed using the 97 overlapping targets in the training and external validation sets **(F)** ROC curve of the optimal Ridge model in the external validation set, with an AUC of 0.956.

At the bulk RNA level, 130 machine learning algorithm combinations were constructed using the 97 overlapping targets, with the TCGA-PRAD cohort serving as the training set and GSE46602 and GSE70768 serving as external validation sets. PCA before and after batch correction showed improved consistency in data distribution between GSE46602 and GSE70768 following correction (Figures 2A–D). These external GEO cohorts were used only for performance evaluation and were not involved in feature selection or model fitting.

Performance evaluation revealed variability among different algorithm combinations in both the training and external validation sets. Among them, the Ridge regression-based model showed the best overall performance, achieving an AUC of 0.956 in the external validation set (Figures 2E,F). These findings indicate that the expression pattern of the overlapping targets showed discriminatory ability between PCa and non-tumor prostate tissues in the analyzed cohorts. However, the high AUC value should be interpreted cautiously, as the modeling strategy was intended for candidate gene prioritization rather than for predicting arecoline exposure, future PCa risk, or clinical diagnostic performance. Candidate genes supported by the top-performing bulk RNA model set were therefore used only to define a preliminary candidate core gene set for subsequent cross-omics integration. In addition to AUC, accuracy, precision, recall, and F1-score were calculated for the top-performing model. Using a classification threshold determined in the TCGA-PRAD training cohort, the model achieved an accuracy of 0.968, precision of 0.996, recall of 0.969, and F1-score of 0.982 in the training cohort. In the combined external GEO validation cohort, the accuracy, precision, recall, and F1-score were 0.775, 0.745, 0.994, and 0.852, respectively. Detailed dataset-specific metrics are provided in Supplementary Table 3.

### 3.3 Identification of key epithelial cell subpopulations by single-cell transcriptomics and core gene screening at the single-cell level

**FIGURE 3:**
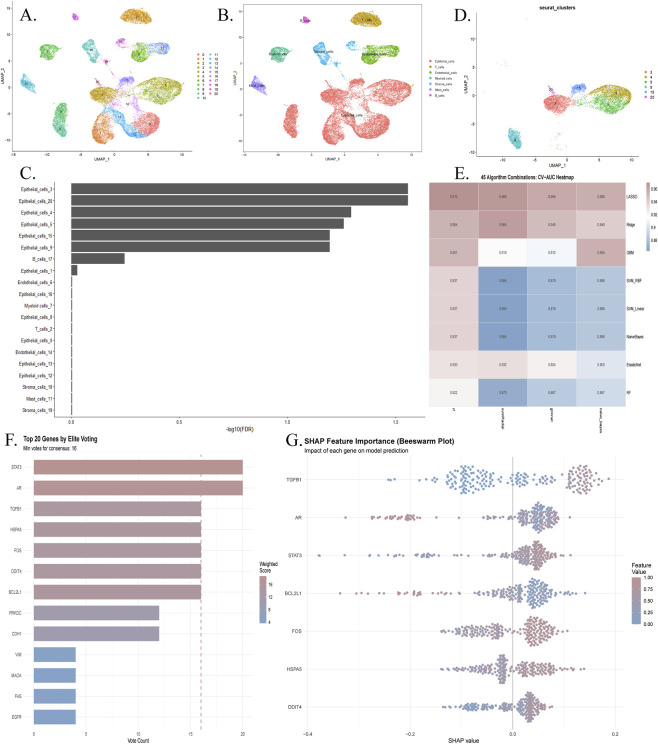
Single-cell transcriptomic analysis identifies key epithelial cell subpopulations and performs core gene screening at the single-cell level **(A)** UMAP plot of the GSE141445 single-cell transcriptomic dataset, showing 21 identified cell clusters **(B)** UMAP plot after cell type annotation, including epithelial cells, T cells, endothelial cells, myofibroblasts, myeloid cells, stromal cells, and B cells **(C)** SeismicGWAS analysis showing the association strength between different cell subpopulations and prostate cancer genetic risk, with several epithelial subpopulations showing the strongest enrichment signals **(D)** UMAP plot of the epithelial subpopulations with the strongest GWAS associations after extraction and re-visualization **(E)** CV-AUC heatmap of 45 machine learning algorithm combinations constructed within the key epithelial subpopulations **(F)** Bar plot showing high-frequency candidate genes identified by the elite voting strategy **(G)** SHAP beeswarm plot showing the direction and magnitude of the contribution of key candidate genes to model prediction.

After quality control, normalization, Harmony-based batch correction, dimensionality reduction, and clustering of the GSE141445 single-cell RNA-seq dataset, a total of 21 cell clusters were identified (Figure 3A). These clusters were further annotated into seven major cell types, including epithelial cells, T cells, endothelial cells, myofibroblasts, myeloid cells, stromal cells, and B cells (Figure 3B).

SeismicGWAS analysis integrating PCa GWAS data (ieu-b-85) with the single-cell transcriptomic profiles showed that several epithelial subpopulations were most strongly associated with PCa genetic risk, including epithelial_cells_3, epithelial_cells_20, epithelial_cells_4, epithelial_cells_5, epithelial_cells_15, and epithelial_cells_9 (Figure 3C). These top-associated epithelial subpopulations were extracted and re-visualized by UMAP, revealing a clear local distribution pattern and providing a basis for focused analysis of intratumoral epithelial heterogeneity and core gene screening (Figure 3D).

Metacell pseudo-bulk expression matrices were then constructed from these key epithelial subpopulations using hdWGCNA, and 45 machine learning algorithm combinations were evaluated based on the overlapping targets. The CV-AUC heatmap showed heterogeneous performance across models, with several combinations built on LASSO, Ridge, and GBM exhibiting relatively favorable predictive ability (Figure 3E). Using an elite voting strategy, high-frequency candidate genes were identified, among which STAT3, AR, TGFB1, HSPA5, FOS, and DDIT4 ranked highly, with AR consistently retained among the top-voted genes (Figure 3F). SHAP analysis further indicated that AR made a prominent contribution to the model output, supporting its prioritization as a candidate gene within epithelial cell populations associated with PCa genetic risk (Figure 3G).

### 3.4 Multidimensional validation of AR expression and functional state analysis in PCa

**FIGURE 4:**
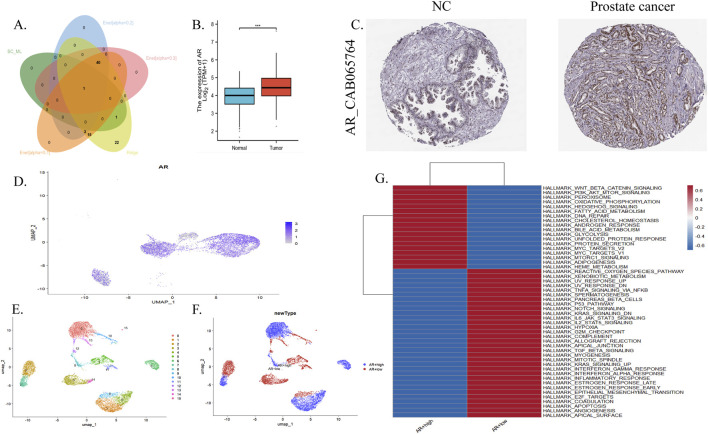
Cross-level identification of AR, multidimensional validation of its expression, and functional state analysis in prostate cancer **(A)** Intersection analysis of candidate genes derived from top-performing bulk RNA models and single-cell machine learning, identifying AR as the only consensus candidate under the predefined cross-level integration strategy **(B)** Differential expression of AR between normal prostate tissues and prostate cancer tissues in the TCGA-PRAD cohort **(C)** Immunohistochemical staining of AR in normal prostate and prostate cancer tissues from the Human Protein Atlas (HPA) database **(D)** UMAP plot showing AR expression distribution in key epithelial cell subpopulations **(E)** UMAP plot of further subclustering of the key epithelial cell populations **(F)** UMAP plot showing the distribution of AR-high and AR-low cells defined by the median AR expression level **(G)** GSVA heatmap comparing pathway activities between AR-high and AR-low epithelial cells. AR-high cells were enriched in WNT/β-catenin signaling, PI3K/AKT/mTOR signaling, mTORC1 signaling, MYC targets, androgen response, glycolysis, fatty acid metabolism, and oxidative phosphorylation, whereas AR-low cells were relatively enriched in p53 pathway, IL6/JAK/STAT3 signaling, TNFα signaling via NFκB, hypoxia, TGF-β signaling, epithelial–mesenchymal transition, and multiple immune/inflammatory pathways.

To further identify a cross-level consensus core gene, the consensus candidate genes derived from single-cell machine learning were intersected with feature genes retained by top-performing models at the bulk RNA level. Importantly, AR was not predefined as the target gene, and all overlapping candidate genes were evaluated under the same screening and integration strategy. Alternative candidates identified in the single-cell voting analysis, including STAT3, TGFB1, HSPA5, FOS, and DDIT4, as well as PPI-ranked genes such as FN1 and MMP2, were also considered during the integration process. However, these genes were not simultaneously retained across the bulk RNA and single-cell screening layers under the predefined cross-level consensus criterion. AR emerged as the only gene consistently retained across multiple optimal models and the single-cell screening results, indicating a high degree of consistency and robustness (Figure 4A).

In the TCGA-PRAD dataset, AR mRNA expression was significantly higher in PCa tissues than in normal prostate tissues (Figure 4B). Consistently, immunohistochemical data from the HPA database showed strong nuclear staining of AR in PCa tissues, whereas staining intensity was relatively weaker in normal prostate tissues, supporting the aberrantly elevated expression of AR at both the transcript and protein levels (Figure 4C).

At the single-cell level, AR was broadly expressed within epithelial subpopulations significantly associated with PCa genetic risk (Figure 4D). Further subclustering of these key epithelial cell populations revealed marked intrapopulation heterogeneity (Figure 4E). Cells were then divided into AR-high and AR-low groups according to the median AR expression level, and UMAP visualization showed distinct spatial distributions between the two groups (Figure 4F). GSVA demonstrated that AR-high cells were enriched for proliferation- and metabolic reprogramming-related pathways, including WNT/β-catenin signaling, PI3K/AKT/mTOR signaling, mTORC1 signaling, MYC targets, androgen response, glycolysis, fatty acid metabolism, and oxidative phosphorylation. In contrast, AR-low cells were relatively enriched for p53 pathway, IL6/JAK/STAT3 signaling, TNFα signaling via NFκB, hypoxia, TGF-β signaling, epithelial–mesenchymal transition, and several inflammation/immune-related pathways (Figure 4G). These findings suggest that higher AR expression in key epithelial subpopulations is associated with a more active tumor-related metabolic and proliferative state.

### 3.5 Molecular simulation of arecoline–AR structural compatibility and expression-level validation of AR response

**FIGURE 5:**
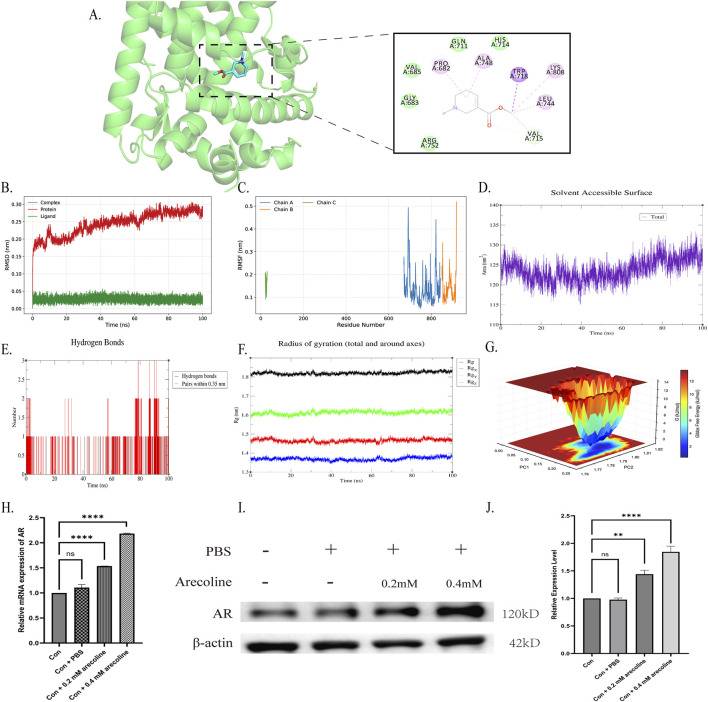
Molecular simulation of arecoline–AR structural compatibility and expression-level validation of AR response **(A)** Three-dimensional docking conformation and two-dimensional interaction diagram of the modeled arecoline–AR complex. The predicted interaction pattern suggests that arecoline may interact with AR through Pi-Sigma, Alkyl/Pi-Alkyl, carbon hydrogen bond, and van der Waals contacts **(B)** RMSD trajectory of the arecoline–AR complex during the 100-ns molecular dynamics simulation **(C)** RMSF analysis of residues in the complex **(D)** Solvent-accessible surface area (SASA) profile of the complex **(E)** Hydrogen bond number during the simulation **(F)** Radius of gyration (Rg) of the complex **(G)** Free energy landscape (FEL) of the arecoline–AR complex **(H)** RT-qPCR quantification of AR mRNA expression in LNCaP cells after treatment with different concentrations of arecoline **(I)** Western blot bands of AR protein expression in LNCaP cells treated with PBS, 0.2 mM arecoline, and 0.4 mM arecoline **(J)** Densitometric quantification of Western blot results. Data are presented as mean ± SD. ns, not significant; **P < 0.01; ****P < 0.0001.

Molecular docking showed that arecoline could be accommodated within a local binding pocket of AR, suggesting structural compatibility between the ligand and the receptor at the computational level. The optimal docking conformation was further analyzed in Discovery Studio, revealing that the interaction between arecoline and AR involved residues PRO682, GLY683, VAL685, GLN711, HIS714, VAL715, TRP718, LEU744, ALA748, ARG752, and LYS808. Specifically, TRP718 formed a Pi-Sigma interaction with the ligand; PRO682, ALA748, LEU744, and LYS808 contributed Alkyl/Pi-Alkyl hydrophobic interactions; VAL715 formed a carbon hydrogen bond; and GLN711, HIS714, VAL685, GLY683, and ARG752 provided supportive van der Waals contacts (Figure 5A).

Further 100-ns molecular dynamics simulations showed that RMSD, RMSF, SASA, hydrogen bond number, and radius of gyration remained within relatively stable ranges throughout the simulation (Figures 5B–F), while the free energy landscape displayed a concentrated low-energy basin (Figure 5G), indicating that the arecoline–AR complex maintained favorable conformational stability under dynamic conditions. MM/PBSA analysis yielded a binding free energy of −23.04 kcal/mol (Table 2). These findings provide computational support for a plausible and relatively stable modeled arecoline–AR interaction. However, docking, molecular dynamics, and MM/PBSA analyses should be interpreted as structural modeling evidence only and cannot be regarded as definitive proof of direct biochemical binding, intracellular target engagement, or *in vivo* interaction.

**TABLE 2:** MM/PBSA binding free energy components of the arecoline–AR complex.

Component	Energy (kcal/mol)	Std. Dev	Meaning
Delta E_vdw	−27.52	1.92	Van der waals interaction
Delta E_ele	−1	0.89	Electrostatic interaction
Delta G_polar	9.23	1	Polar solvation energy
Delta G_nonpolar	−3.77	0.15	Non-polar solvation energy
Delta G_bind (total)	−23.04	1.76	Total binding free energy

To provide preliminary expression-level validation of the bioinformatic findings, LNCaP cells were treated with 0.2 mM and 0.4 mM arecoline for 24 h, followed by assessment of AR mRNA and protein expression. RT-qPCR showed that AR mRNA expression increased after arecoline treatment compared with the control group and the PBS vehicle control, with a more pronounced increase observed in the 0.4 mM group, whereas no significant difference was found between the PBS and control groups (Figure 5H). Western blot analysis further demonstrated that arecoline treatment upregulated AR protein expression, again with a more marked increase in the high-dose group; densitometric quantification was consistent with the immunoblot band patterns (Figures 5I,J). Together, these findings suggest that arecoline exposure was associated with increased AR mRNA and protein expression in LNCaP cells under the present experimental conditions. However, these expression-level results do not establish direct AR binding or downstream functional activation of AR signaling.

## 4 Discussion

Arecoline, the principal alkaloid of areca nut, is one of the major bioactive components responsible for the toxicological effects of areca-related products (Huang et al., 2024; Wang Z. et al., 2024). Because areca nut and its derived products are widely chewed or consumed in certain Asian regions and other specific populations worldwide, arecoline should be regarded not only as a commonly encountered environmental toxicant but also as a compound with clear oral and food-related exposure relevance (Huang et al., 2024; Mehrtash et al., 2017; Gupta and Warnakulasuriya, 2002). Increasing evidence has linked arecoline and areca-related exposure to a variety of adverse health outcomes, and their carcinogenic or tumor-promoting roles have been more extensively investigated in oral cancer and some upper gastrointestinal malignancies (Ko et al., 2023; Guha et al., 2014; Akhtar, 2013; Humans Research International Agency, 2004). However, compared with the growing body of evidence in oral and digestive tract tumors, the potential role of arecoline in prostate cancer remains insufficiently explored. Existing studies have only suggested that arecoline may influence normal prostate cells and prostate cancer cells through ROS-related and cell-cycle-related mechanisms, while whether it participates in the dysregulation of key PCa-associated molecular programs, especially AR-related signaling, has remained largely unclear (Shih et al., 2020; Saha et al., 2011). Thus, the novelty of the present study does not lie in establishing arecoline as a confirmed PCa risk factor, but in providing an exploratory systems-level framework to evaluate whether arecoline-related target networks converge on PCa-relevant, AR-centered molecular programs. In this context, exposure relevance is also an important consideration when interpreting the present *in vitro* findings. The concentrations used in LNCaP cells, 0.2 and 0.4 mM, correspond to approximately 31.0 and 62.1 μg/mL arecoline, respectively. These concentrations partially overlap with some reported salivary arecoline levels during areca nut chewing, although other studies have reported substantially lower levels (Cox et al., 2010; Venkatesh et al., 2018). Such discrepancies may reflect differences in areca products, chewing habits, sampling time, saliva flow, analytical methods, and population characteristics. More importantly, salivary exposure cannot be directly equated with systemic circulation or prostate tissue exposure. Therefore, our *in vitro* experiments should be interpreted as an exploratory high-exposure model designed to test the molecular responsiveness of AR to arecoline, rather than as a direct simulation of physiological prostate exposure. Future pharmacokinetic, biomonitoring, and tissue-distribution studies are needed to define biologically realistic exposure windows for prostate-related toxicological assessment.

Prostate cancer is one of the most common malignancies in men worldwide and remains a major cause of cancer-related morbidity and mortality (Bray et al., 2024). Although PSA screening, radical surgery, radiotherapy, and androgen deprivation therapy have improved outcomes in selected patients, advanced disease, particularly castration-resistant prostate cancer, still poses major clinical challenges because of progression, recurrence, and therapeutic resistance (Raychaudhuri et al., 2025). As a central driver of prostate cancer biology, AR is critically involved in tumor initiation, sustained proliferation, metastatic progression, and treatment resistance (Balk, 2014). Therefore, identifying upstream exogenous factors that may contribute to abnormal AR activation is of considerable importance for understanding PCa pathogenesis, especially in the context of environmentally relevant endocrine-disrupting or hormone-interfering compounds. In this context, the current evidence linking arecoline to PCa is still very limited and fragmented (Lacouture et al., 2022). Our study was designed to address this gap by integrating network toxicology, bulk RNA machine learning, single-cell transcriptomics, SeismicGWAS, molecular simulation, and *in vitro* validation, thereby providing a more systematic framework for understanding the potential molecular association between arecoline exposure and prostate cancer. Importantly, the present findings should not be interpreted as evidence that arecoline causes prostate cancer or directly promotes PCa progression in humans. Instead, our results support a hypothesis-generating model in which arecoline-related target networks converge on AR-associated transcriptional and cellular states relevant to PCa biology. Although AR was consistently prioritized across network topology, bulk RNA machine learning, single-cell screening, and molecular simulation, these results indicate candidate molecular relevance rather than confirmed causal mediation. Therefore, the current study should be viewed as an exploratory systems toxicology framework for identifying potential molecular links between arecoline exposure and PCa-related alterations.

Methodologically, this study did not rely on a single database-based prediction or a single transcriptomic analysis, but instead established a multi-layered framework integrating network toxicology, bulk RNA machine learning, single-cell transcriptomics, and SeismicGWAS to identify key molecules linking arecoline and PCa from tissue-level, cell-subpopulation-level, and disease-genetic perspectives. We first identified 97 overlapping targets between arecoline and PCa, and these genes were mainly enriched in apoptosis-, p53-, TNF-, HIF-1-, IL-17-, and MAPK-related pathways, suggesting that the potential arecoline–PCa connection may involve tumor cell survival, stress adaptation, and inflammatory signaling. We acknowledge that apoptosis-, p53-, and MAPK-related pathways are broadly involved in many cancer types and should not be considered specific to arecoline exposure or PCa alone. In the present study, these enrichment results were interpreted as pathway-level functional context rather than as definitive mechanistic evidence. Their relevance to the arecoline–PCa framework lies in the fact that arecoline has been reported to influence ROS production and cell-cycle regulation in prostate-related experimental models, while apoptosis regulation, p53-associated stress responses, and MAPK signaling are closely related to cellular stress adaptation, survival, and proliferation in PCa biology. Therefore, these pathways provide a biologically plausible background for prioritizing candidate genes, but further functional studies are required to determine whether they are directly modulated by arecoline in prostate cancer cells. Furthermore, we recognize that network toxicology approaches are inherently influenced by the completeness and annotation quality of public databases. Literature-driven bias may also lead to the overrepresentation of well-studied proteins as hub nodes in PPI networks. Therefore, the PPI topology and hub-gene ranking were not interpreted as definitive mechanistic evidence. Instead, they were used as an initial prioritization step, and the candidate genes were further evaluated through independent transcriptomic cohorts, single-cell analysis, SeismicGWAS-guided epithelial subpopulation prioritization, protein-expression evidence, molecular simulation, and expression-level experimental validation. At the bulk RNA level, the top-performing model showed discriminatory ability between PCa and non-tumor prostate tissues, supporting the use of these genes for candidate prioritization at the tissue level. We then integrated single-cell transcriptomics with SeismicGWAS and found that several epithelial subpopulations were most strongly associated with PCa genetic risk. Within these key epithelial cell populations, metacell-based machine learning and SHAP analysis consistently highlighted AR as an important candidate. Finally, by integrating the bulk RNA results, single-cell screening, and PPI network information, AR emerged as the only consensus core candidate gene. Together, these findings suggest that the link between arecoline and PCa is unlikely to be an isolated result from a single analytical layer, but rather may reflect a stable cross-level association involving network topology, tissue-level expression patterns, and disease-relevant epithelial cell states.

AR is one of the most representative master regulators in prostate biology, playing indispensable roles in maintaining epithelial differentiation, secretory function, and tissue homeostasis (Balk, 2014). In prostate cancer, AR remains a central molecular driver throughout tumor initiation, sustained proliferation, metastatic dissemination, and therapeutic resistance (Balk, 2014). Accordingly, any exogenous factor capable of influencing AR expression, activation status, or downstream transcriptional programs may have important implications for PCa biology (Lacouture et al., 2022). In the present study, AR was not merely identified because it is a well-known PCa-related gene. No candidate gene was manually preselected or weighted during the screening process. Instead, AR was repeatedly prioritized across multiple analytical layers and ultimately retained as the only consensus candidate shared by PPI topology, tissue-level machine learning, and single-cell screening. Other candidates, including STAT3, TGFB1, HSPA5, FOS, DDIT4, FN1, and MMP2, were also evaluated, but they did not show the same level of cross-layer consistency under the predefined integration strategy. This does not exclude their potential biological relevance, but indicates that AR showed the strongest consensus support in the present exploratory framework. This consistency strongly suggests that AR may serve as a key molecular interface between arecoline exposure and prostate cancer-related molecular alterations. Furthermore, the single-cell analysis showed that AR-high epithelial cells were enriched for androgen response, PI3K/AKT/mTOR signaling, mTORC1 signaling, MYC targets, and several metabolism-related pathways, indicating that higher AR expression may be associated with a more active tumor-promoting functional state. Thus, in the context of our study, AR should be viewed not only as a classical driver of PCa progression but also as a plausible mechanistic entry point through which arecoline may influence PCa-associated molecular programs.

To further support the above multi-omics findings, we performed molecular simulation and preliminary *in vitro* expression-level validation. Molecular docking suggested that arecoline could be accommodated within a local binding pocket of AR and form several non-covalent contacts, while the subsequent 100-ns molecular dynamics simulation and MM/PBSA analysis provided computational support for the structural stability of the modeled arecoline–AR complex. However, these results should be interpreted as supportive structural modeling evidence rather than direct biochemical proof of arecoline–AR binding. In parallel, our LNCaP cell experiments showed that arecoline treatment increased AR mRNA and protein expression, particularly at the higher concentration. These findings indicate that AR expression is responsive to arecoline exposure under the present experimental conditions, but they do not demonstrate direct target engagement, canonical AR transcriptional activation, or downstream malignant phenotypes. Therefore, the molecular simulation and *in vitro* data should be viewed as preliminary support for prioritizing AR as a candidate molecular node, rather than as definitive mechanistic validation.

From a broader perspective, the implications of this study extend beyond prostate cancer biology alone and are also relevant to epidemiology, environmental toxicology, and food-related toxicology. Because areca-related products are widely used in some populations, arecoline exposure has a substantial real-world public health background, making evaluation of its long-term health risks epidemiologically meaningful (Lacouture et al., 2022; Feijo et al., 2025; Huang et al., 2024). Most previous toxicological studies on arecoline have focused on oral mucosal injury, oral carcinogenesis, or upper digestive tract malignancies (Arora and Squier, 2019; Wang Z. et al., 2024; Ko et al., 2023). Our findings suggest that its potential impact may extend to the prostate, a hormone-responsive organ, and may involve dysregulation of AR-centered molecular programs (Lacouture et al., 2022). This expands the current understanding of the toxicological spectrum of arecoline and suggests that its risk profile may not be confined to local exposure sites alone. In addition, given its clear oral and food-related exposure relevance, our results indicate that food toxicology assessments of arecoline should perhaps move beyond conventional local toxicity or classical carcinogenic endpoints and pay more attention to its potential effects on endocrine-related signaling networks and distant organ molecular homeostasis.

Several strengths of this study should be highlighted. First, this study was not based on a single computational approach, but integrated network toxicology, bulk RNA machine learning, single-cell transcriptomics, SeismicGWAS, molecular simulation, and preliminary *in vitro* validation, which strengthened the internal consistency of candidate prioritization. Second, by combining single-cell analysis with GWAS-guided prioritization, we were able to evaluate candidate genes not only at the tissue level but also within epithelial subpopulations with potential relevance to PCa genetic susceptibility, thereby improving the cellular specificity of the analysis. Third, AR was repeatedly prioritized across multiple analytical layers, supporting its consistency as a candidate molecular node. Finally, the study extended beyond purely bioinformatic inference by incorporating structural simulation and expression-level experimental validation, providing preliminary support for the biological relevance of AR in this exploratory framework.

These strengths notwithstanding, the machine learning results should be interpreted with caution. Although the use of multiple algorithms enabled a broad comparison of candidate prioritization strategies, the construction of a large number of models may introduce overfitting and model-selection bias, particularly because a formal nested cross-validation framework was not applied. In the present study, the models were used to prioritize candidate genes showing transcriptomic relevance across bulk and single-cell analytical layers, rather than to establish a clinical diagnostic tool or an exposure-related risk prediction model. At the single-cell level, repeated cross-validation within metacell-based epithelial subpopulations provided an internal performance estimate, but it did not replace independent external validation. Therefore, the high AUC values should be considered supportive for candidate prioritization, but not as definitive evidence of clinical predictive robustness.

Several limitations should also be acknowledged. First, this study was exploratory and hypothesis-generating in nature. The current evidence does not establish a causal relationship between arecoline exposure and PCa development or progression. Second, the initial target space was derived partly from public databases and prediction tools, which may introduce false positives, incomplete target coverage, literature-driven bias, and overrepresentation of well-studied proteins. Therefore, database-derived targets were used only as a hypothesis-generating feature space and were further prioritized through multi-step cross-level analyses rather than interpreted as experimentally confirmed direct targets of arecoline. Third, although relatively consistent results were obtained across several public datasets, the bulk RNA and single-cell analyses remain retrospective bioinformatic investigations and may still be affected by sample heterogeneity, dataset-specific differences, and model-selection bias. Fourth, the current experimental validation demonstrated AR upregulation at the mRNA and protein levels, but did not assess canonical AR transcriptional activity or malignant phenotypes such as proliferation, invasion, migration, apoptosis resistance, or treatment resistance. Fifth, although molecular docking and molecular dynamics simulations suggested structural compatibility between arecoline and AR, these computational approaches cannot substitute for direct biochemical binding assays. In addition, replicate MD simulations were not performed; therefore, the MD results should be interpreted as supportive computational evidence rather than definitive proof of dynamic stability under all simulation conditions. Finally, although the selected *in vitro* concentrations were contextualized against previous prostate cell studies and reported salivary exposure ranges, our study lacks pharmacokinetic data, prostate tissue-distribution evidence, *in vivo* animal evidence, and population-based epidemiological validation. Therefore, future studies incorporating functional experiments, direct target-engagement assays, animal models, exposure assessment, and epidemiological investigations are needed to further test the hypotheses proposed here.

## 5 Conclusion

In conclusion, this study integrated network toxicology, bulk RNA machine learning, single-cell transcriptomics, SeismicGWAS, molecular simulation, and *in vitro* validation to explore the potential molecular association between arecoline exposure and PCa-related alterations. AR emerged as a cross-level candidate gene consistently supported by network, tissue-level, and single-cell analyses. Molecular simulation suggested structural compatibility between arecoline and AR, while *in vitro* experiments showed that arecoline increased AR mRNA and protein expression in LNCaP cells. These findings do not establish a causal relationship between arecoline exposure and PCa, but support AR as a plausible candidate molecular node for future mechanistic, exposure-focused, and epidemiological studies. Beyond their relevance to prostate cancer biology, these findings also support further investigation of arecoline within both environmental toxicology and food-related or oral exposure toxicology contexts.

## Data Availability

Publicly available datasets were analyzed in this study. This data can be found here: The datasets analysed during the current study are publicly available from public repositories: TCGA Prostate Adenocarcinoma data via the GDC portal (https://portal.gdc.cancer.gov/), GEO datasets via NCBI (accession numbers GSE141445, GSE46602 and GSE70768).

## Supplementary material

SUPPLEMENTARY TABLE 1 Target genes of arecoline.

SUPPLEMENTARY TABLE 2 Target genes of prostate cancer.

SUPPLEMENTARY TABLE 3 Performance metrics of the top-performing bulk RNA model in the training and external validation datasets.

SUPPLEMENTARY TABLE 4 Databases and computational tools used in this study.
